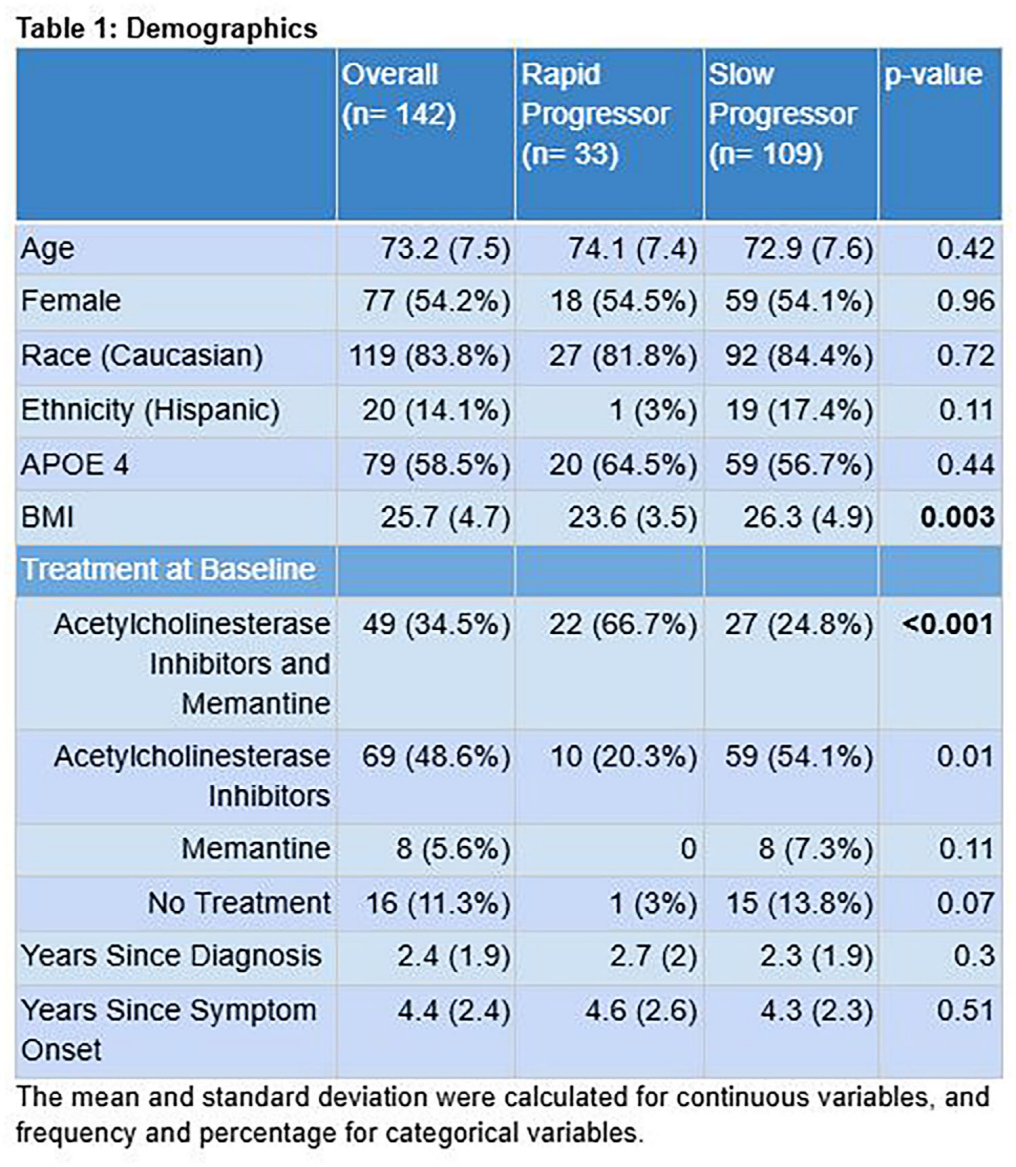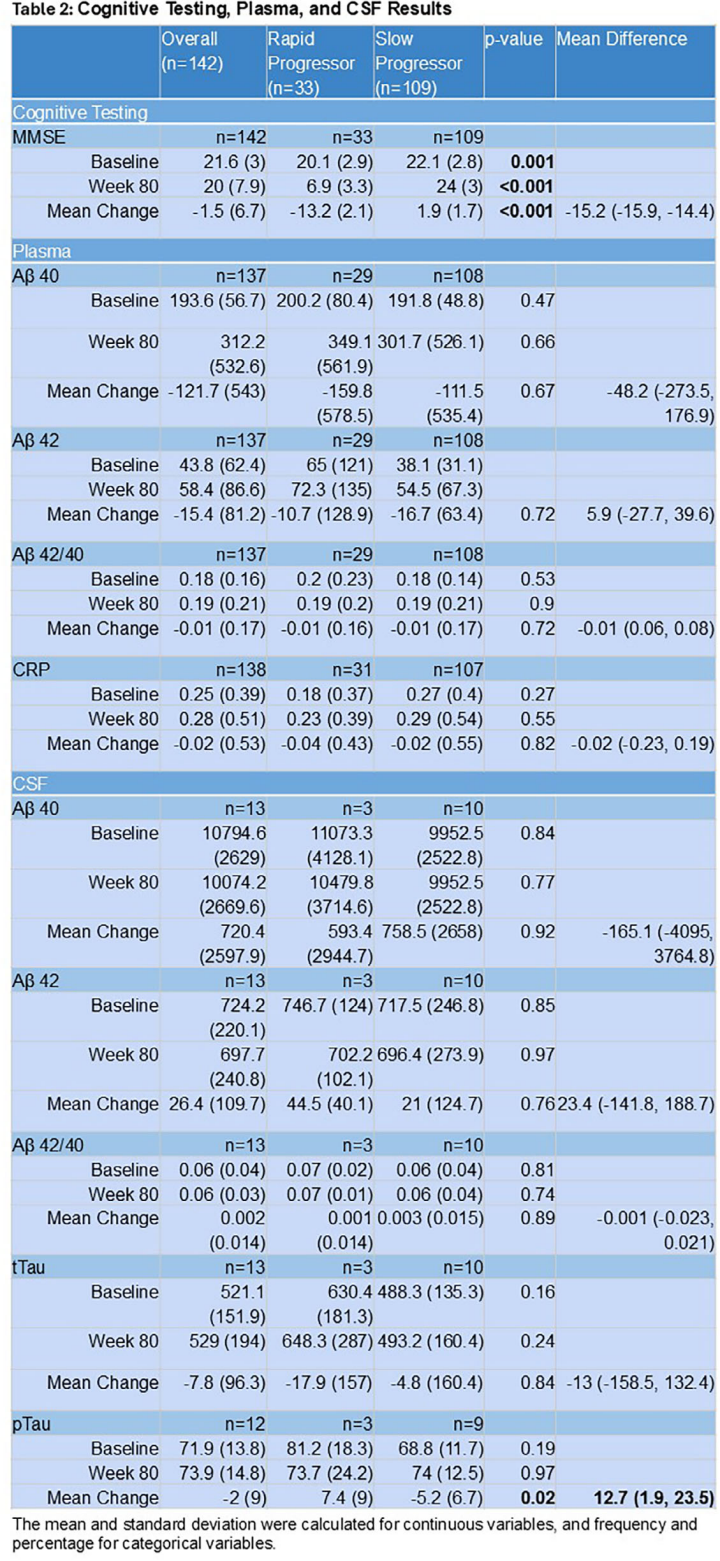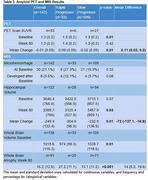# Rapid Progressors in Alzheimer's Disease: Implications for Symptomatic Clinical Trial Design

**DOI:** 10.1002/alz70856_101271

**Published:** 2025-12-24

**Authors:** Madison Shyer, Kristofer Harris, Dulin Wang, Yejin Kim, Xiaoqian Jiang, Paul E Schulz

**Affiliations:** ^1^ UTHealth Houston, Houston, TX, USA; ^2^ John P. and Kathrine G. McGovern Medical School at UTHealth, Houston, TX, USA; ^3^ The University of Texas Health Science Center at Houston, Houston, TX, USA; ^4^ University of Texas Health Science Center at Houston, Houston, TX, USA

## Abstract

**Background:**

Previous clinical trials for Alzheimer's disease (AD) have identified different progression patterns among patients: some showing minimal cognitive change (slow progressors, SPs) and others experiencing rapid decline (rapid progressors, RPs). This study aims to explore the differences between RPs and SPs to better understand their impact on clinical trial outcomes, drug effects, and caregiving.

**Method:**

RPs and SPs were defined from deidentified, placebo arm data from the EXPEDITION 1 randomized control trial. RPs were defined as patients who declined >10 points on the MMSE while SPs exhibited no decline from screening to week 80. Demographics, MMSE, plasma, CSF, MRI, and PET scan variables were assessed for statistically significant differences.

**Results:**

A total of 142 patients were included, 33 (23.2%, 18 female) RPs and 109 (76.7%, 59 female) SPs. Statistically significant differences between the RPs and SPs included BMI (RP mean 25.7 (SD 4.7) vs SP 26.3 (4.9); *p*‐value: 0.003), treatment with an acetylcholinesterase inhibitor and memantine (34.5% vs 24.8%; *p*‐value: <0.001), baseline MMSE (20.1 (2.9) vs 22.1 (2.8); *p*‐value: 0.001), baseline plasma amyloid beta 42 (65 (121) vs 38.1 (31.1); *p*‐value: 0.03), baseline amyloid PET scan SUVR (1.5 (0.2) vs 1.3 (0.2); *p*‐value: 0.01), whole brain volume at baseline (974 (99.3) vs 1028.7 (118.6); *p*‐value: 0.01), and whole brain atrophy at week 80 (27.2 (11.1) vs 13.1 (11.2); *p*‐value: <0.001). The mean difference between RPs and SPs at week 80 (RPs ‐ SPs) was ‐15.2 (CI: ‐15.9‐ ‐14.4) for MMSE, 12.7 (CI: 1.9‐23.5) for pTAU in the CSF, ‐72 (CI: ‐127.1‐ ‐16.8) for hippocampal volume, and 14.8 (CI: 8.3‐19.6) for whole brain atrophy.

**Conclusions:**

This study reveals significant differences between RPs and SPs in various clinical, imaging, and biochemical markers, highlighting the interplay of factors influencing AD progression. The results show the need for tailored approaches to clinical trials to account for different progression patterns within AD.